# Supplementary material for: Lipid Rafts Interaction of the ARID3A Transcription Factor with EZRIN and G-Actin Regulates B-Cell Receptor Signaling
Source: Diseases. 2021 Mar 20;9(1):22. doi: 10.3390/diseases9010022 (PMC8005928; doi:10.3390/diseases9010022)
Supplement: Supplementary file 1 [file diseases-09-00022-s001.zip › diseases-1126193-supplementary.docx]

Supplemental Figure Legends


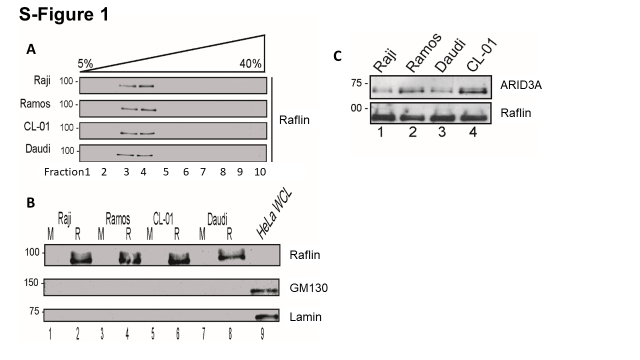


**Figure S1.** Biochemical fractionation indicates that lipid rafts, but not other subcellular compartments, vary among B cell tumors in levels of ARID3A. A, B. Confirmation of lipid raft purity. Raji, Ramos, CL-01 and Daudi cells were subjected to preparation of crude plasma membranes and lipid rafts using sucrose gradient centrifugation and the insolubility of lipid rafts in ice-cold Triton X-100 containing buffer. Fractions were taken from the top of the gradient to the bottom and analyzed by western blot using anti-Raftlin antiserum. To further demonstrate the purity of plasma membranes and lipid rafts, we performed a series of Western assays, shown in panel B. Raji, Ramos, CL-01 and Daudi cells were subjected to preparation of plasma membranes (M) and lipid rafts (R). Whole cell lysates (WCL) from HeLa cells served as control. Reprobing of the same filters with anti-GM-130, a marker for the Golgi membrane, and anti-Lamin-B, a nuclear marker, show that M nor R preparations show detectable levels of these proteins, whereas HeLa whole cell extract served as positive control. C. Lipid rafts from B cell lines contain variant levels of ARID3A. Lipid rafts were purified from Raji, Ramos, CL-01 and Daudi cells, converted to WCLs, and then Western Blotted with anti-ARID3A and then re-probed with Raftlin as a loading control. All results are representative of at least 3 independent experiments.

**
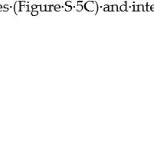

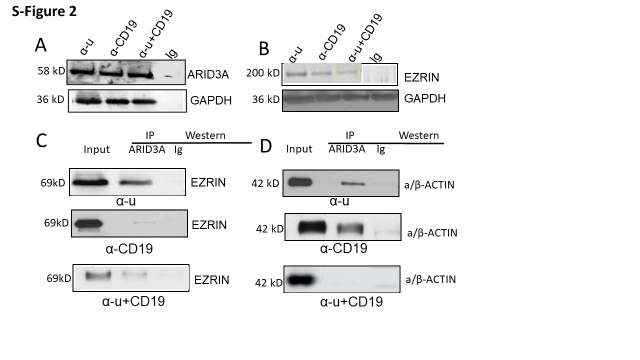
**

**Figure S2. ARID3A interaction with EZRIN and Actin depends on the signaling threshold of the BCR.**  Normal follicular (FO) B cells (~108 cells/dish) were stimulated for 5 min with either 100 ng anti-IgM(α-IgM), or 100 ng α-CD19 or 100 ng α-IgM +100 ng α-CD19. **A, B**. FO B cell whole cell lysates (WCL) were analyzed by Western blotting with anti-ARID3A (A) or by anti-EZRIN (B). GAPDH served as a loading control; input controls (panels 1, 3); Ig-only negative controls (panels 4). Levels of neither ARID3A nor EZRIN were significantly altered under the activation conditions employed. These WCLs were employed as inputs for Co-immunoprecipitation (Co-IP analyses: **C, D.** ARID3A interaction with ERZIN or Actin is dependent on BCR signaling strength. Anti-ARID3A immunoprecipitates of FO B cell WCLs were fractionated on SDS-PAGE and then blotted with either anti-EZRIN (**C**) or anti-αβActin (**D**). Shown are blots representative of 3 independent experiments. **C.** EZRIN Co-IPs with ARID3A modestly under modest signaling condition (α-IgM or α-CD19) but avidly following strong signaling via α-IgM + α-CD19. **D.** Parallel Co-IP analysis of ARID3A with Actin showed the same signal strength dependence: ie, strongest stimulation provided the strongest interaction.

**
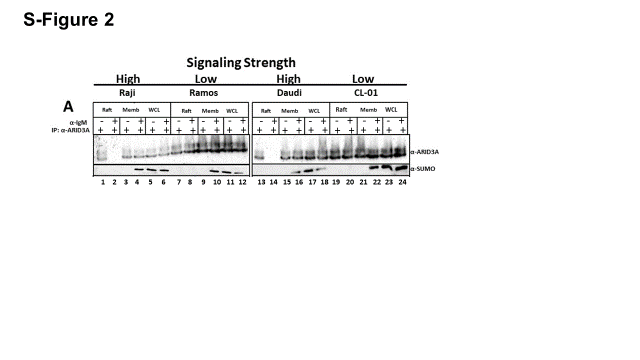
**

**Figure S3. Strength of BCR stimulation leads to differential lipid rafts discharge of ARID3A, but not its interacting partner, SUMO-I.** SUMO-I levels were assessed under conditions in which lipid rafts levels of ARID3A were modulated as described in the text and in Figure 1. **A.** Following moderate stimulation with anti-IgM, ARID3A is discharged from lipid rafts of Raji and Daudi cells (lanes 2 and 14), but not from those of Ramos and CL01 cells (lanes 8 and 20). **B.** ARID3A remains localized within rafts of all cell lines following weak stimulation by anti-CD19 (lanes 2, 8, 14 and 20). **C.** Under the strongest stimulatory condition (anti-IgM + anti-CD19), ARID3A is fully discharged (lanes 2, 8, 14 and 20). **A-C.** No change was observed in the expression levels of SUMO-1, which modifies ARID3A and ARID3C at single K residues (Figure S 5C) and interacts with ARID3A and ARID3C in lipid rafts (22, 33)

**
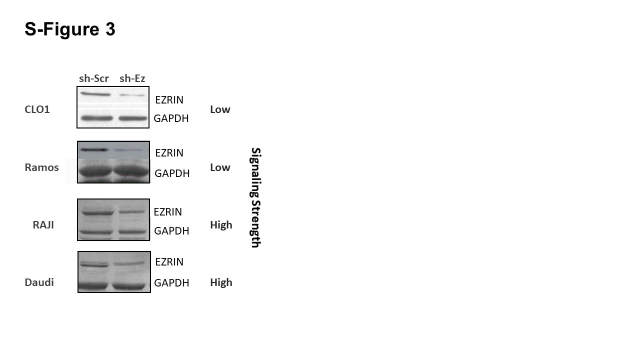
**

**Figure S4. EZRIN retroviral knockdown levels.** Levels of EZRIN were determined following sh-EZRIN and sh-Scrambled transduced lymphoma/leukemia cells via Western blotting as detailed in Methods and Materials. Data shown are representative of three independent experiments.


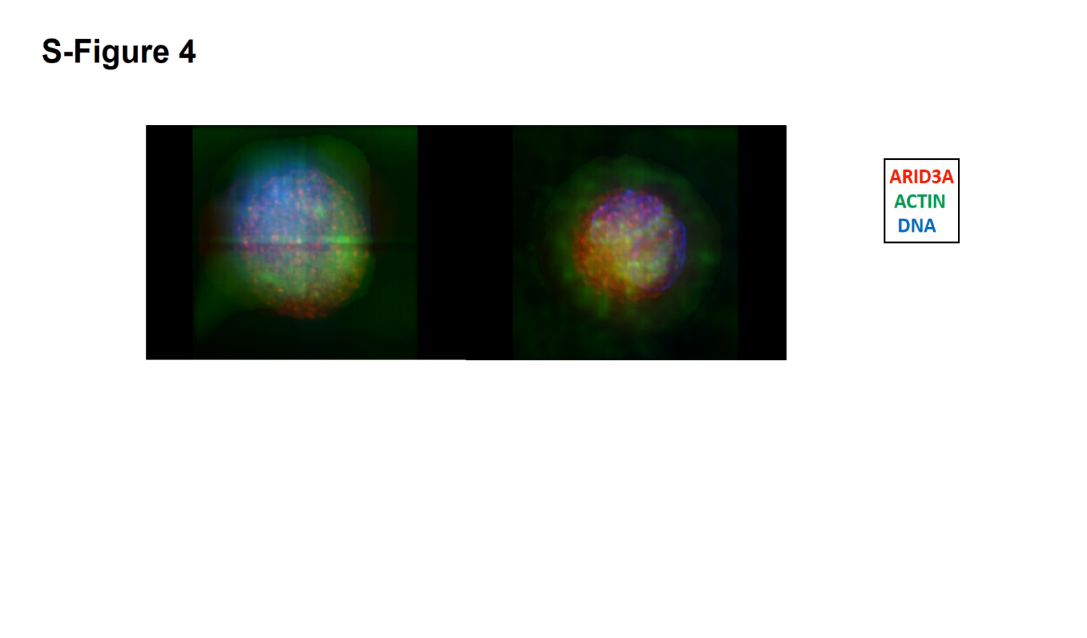

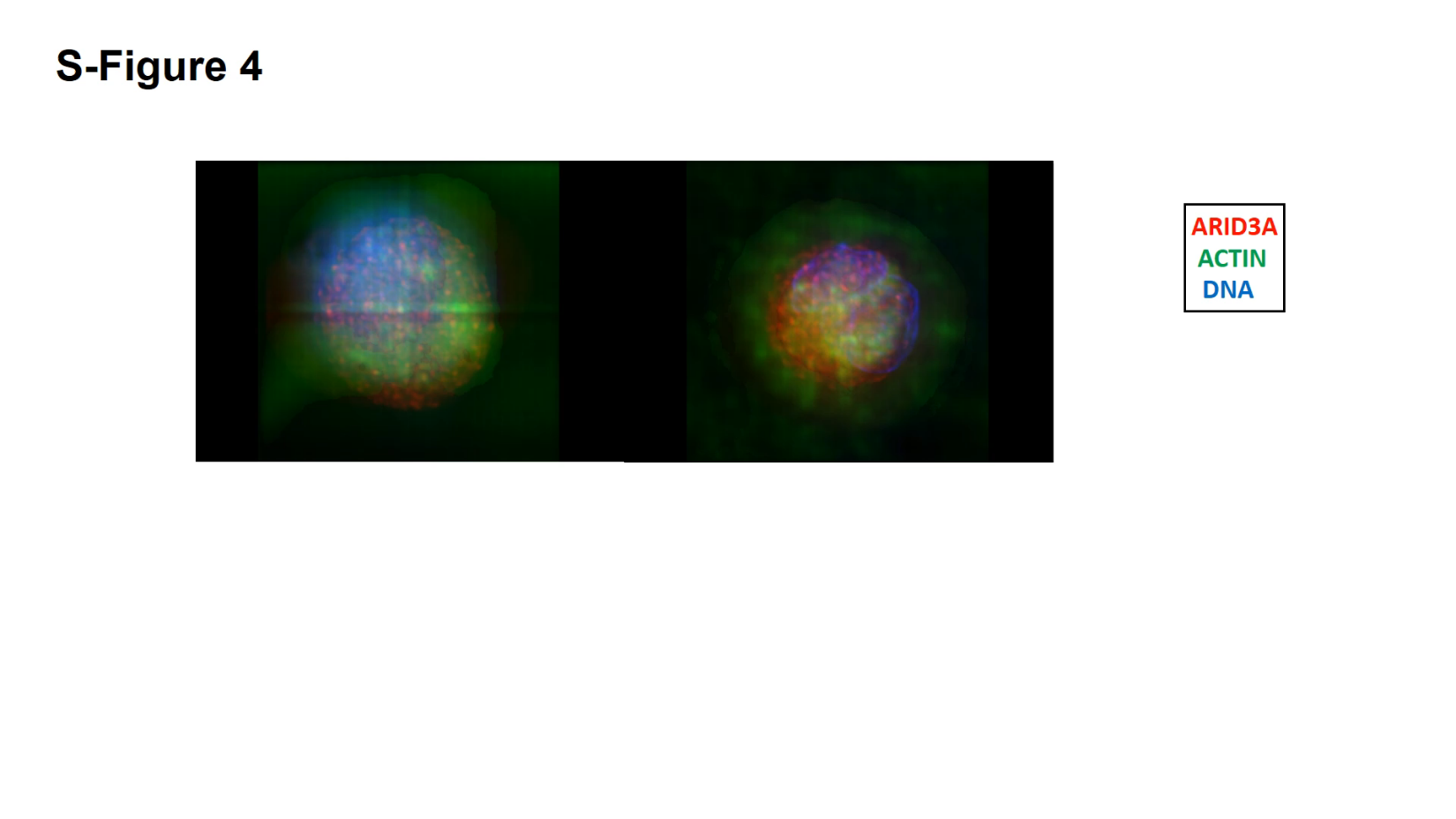


**Figure S5. Computerized 3D reconstruction demonstrate Actin-ARID3A interaction in B lymphocytes.** Murine FO B cells were purified, stained with fluorescent-antibodies and imaged as detailed in Materials and Methods. Significant colocalisation (yellow) was observed for ARID3A (red) and Actin (green). DAPI-stained nuclei are shown in blue.


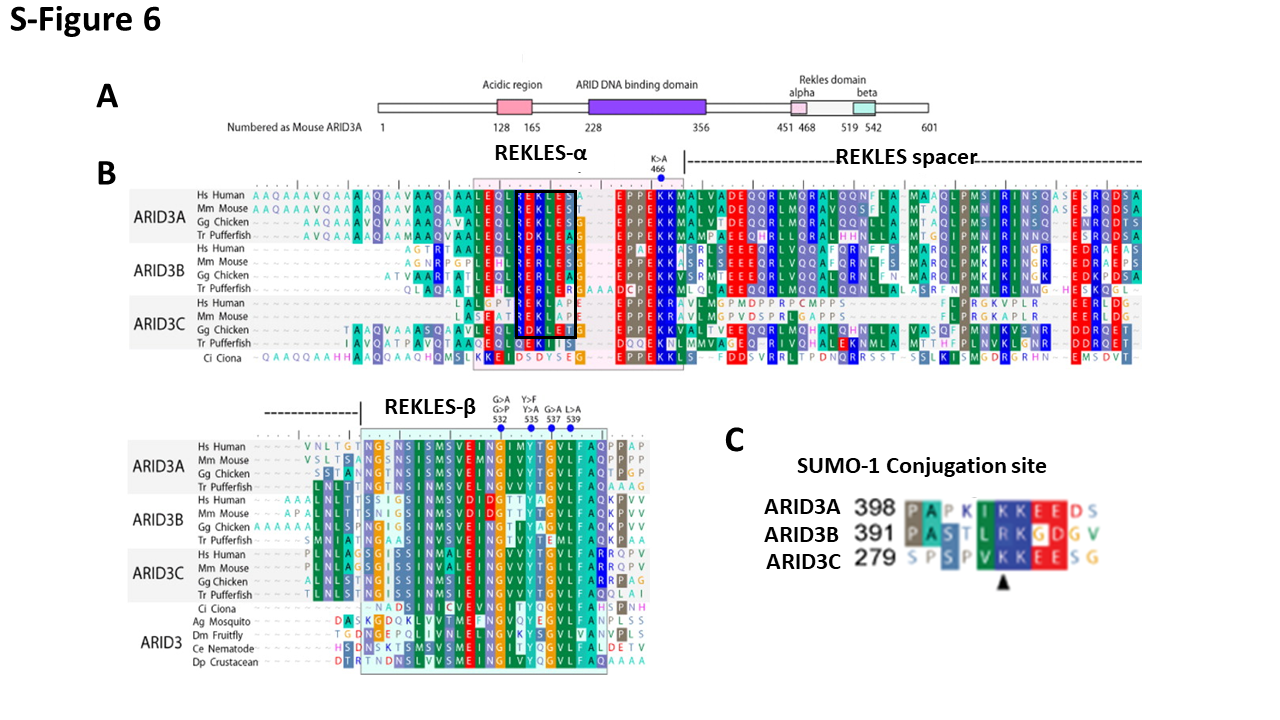


**Figure S6. ARID3 family REKLES domain alignments**. **A.** Schematic of ARID3A with domains denoted in colored boxes, including the Acidic, DNA binding (ARID) and multifunctional homomerization/nuclear export/EZRIN-interacting REKLES domain. **B.** The alignment of ARID3 REKLES-α subdomains of human (*Homo sapiens*), mouse (*Mus musculus*), chicken (*Gallus gallus*), pufferfish (*Takifugu rubripes*), and the urochordate tunicate Ciona (*Ciona intestinalis*) are shown. For the REKLES-β alignment, additional sequences from mosquito (*Anopheles gambiae*), fruit fly (*Drosophila melanogaster*), nematode (*Caenorhabditis elegans*), and the crustacean Daphnia (*Daphnia pulex*) are indicated. Point mutants used in this study are denoted with blue dots above the aligned sequences. **C.** A SUMO-I consensus motif with conjugated K (▲) is located N-terminal to the REKLES domain at the indicated positions for ARID3A and ARID3C. Modified from references 33 and 35.

**
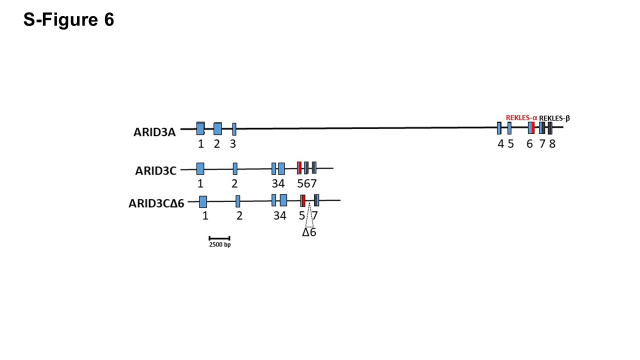
**

**Figure S7. Schematic of ARID3A and ARID3C loci.** Shown in scale (kb marker at bottom) are exons encoding ARID3A, ARID3C, and ARID3C∆6. ARID3C and ARID3C∆6 loci are compressed relatively to that of ARID3A. REKLES-α exons, red; REKLES-β exons, gray. The dotted triangle indicates the alternative pre-mRNA skipping of exon 6.
