# Supplementary figures and images for: Lipid Rafts Interaction of the ARID3A Transcription Factor with EZRIN and G-Actin Regulates B-Cell Receptor Signaling
Source: Diseases. 2021 Mar 20;9(1):22. doi: 10.3390/diseases9010022 (PMC8005928; doi:10.3390/diseases9010022)

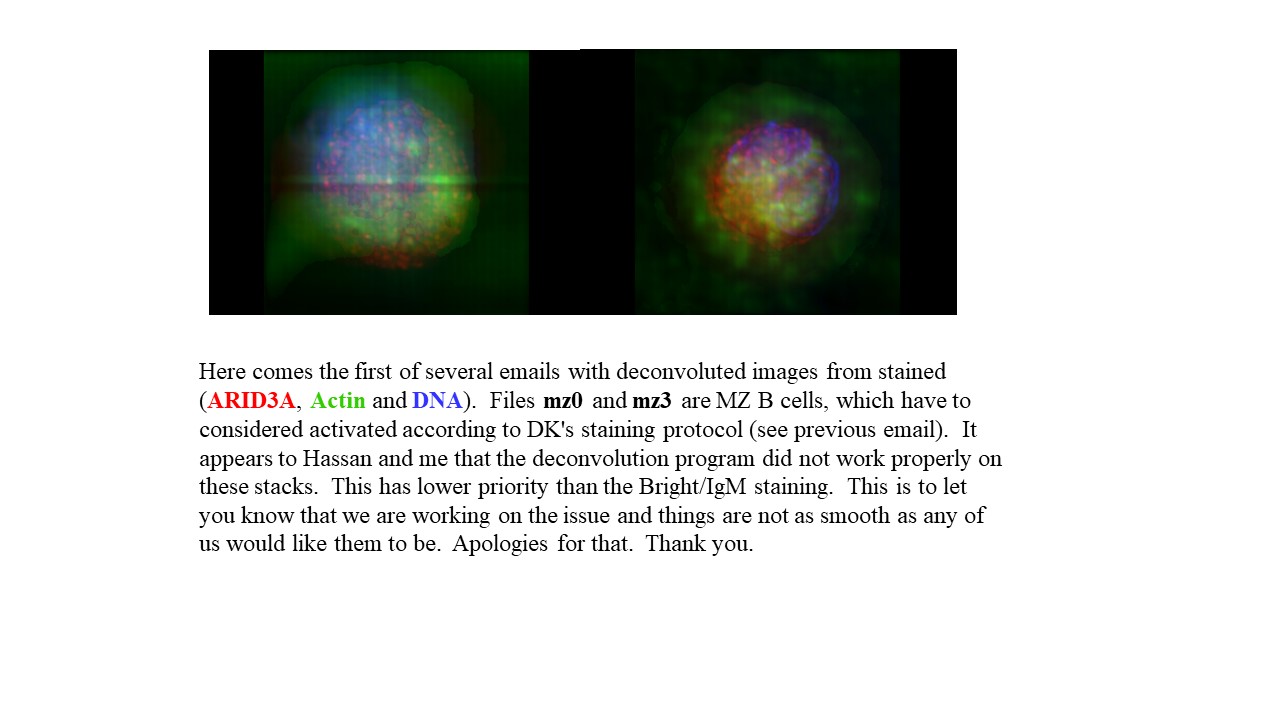

Supplement: Supplementary file 1 [file diseases-09-00022-s001.zip › Video S1, S2.jpg]
